# Chinese medicinal herbs as potential prodrugs for obesity

**DOI:** 10.3389/fphar.2022.1016004

**Published:** 2022-10-03

**Authors:** Siu Kan Law, Yanping Wang, Xinchen Lu, Dawn Ching Tung Au, Wesley Yeuk Lung Chow, Albert Wing Nang Leung, Chuanshan Xu

**Affiliations:** ^1^ Key Laboratory of Molecular Target & Clinical Pharmacology and the State & NMPA Key Laboratory of Respiratory Disease, School of Pharmaceutical Sciences, Fifth Affiliated Hospital, Guangzhou Medical University, Guangzhou, China; ^2^ Faculty of Science and Technology, The Technological and Higher Education Institute of Hong Kong, Hong Kong, Hong Kong SAR, China; ^3^ School of Nursing and Health Studies, Hong Kong Metropolitan University, Hong Kong, Hong Kong SAR, China; ^4^ School of Graduate Studies, Lingnan University, Tuen Mun, Hong Kong SAR, China

**Keywords:** obesity, herbal medicine, prodrug, traditional Chinese medicine (TCM), active ingredient, medicinal plants, bioavailability, nanotechnology

## Abstract

Obesity is a leading worldwide health threat with ever-growing prevalence, it promotes the incidence of various diseases, particularly cardiovascular disease, metabolic syndrome, diabetes, hypertension, and certain cancers. Traditional Chinese Medicine (TCM) has been used to control body weight and treat obesity for thousands of years, Chinese medicinal herbs provide a rich natural source of effective agents against obesity. However, some problems such as complex active ingredients, poor quality control, and unclear therapeutic mechanisms still need to be investigated and resolved. Prodrugs provide a path forward to overcome TCM deficiencies such as absorption, distribution, metabolism, excretion (ADME) properties, and toxicity. This article aimed to review the possible prodrugs from various medicinal plants that demonstrate beneficial effects on obesity and seek to offer insights on prodrug design as well as a solution to the global obesity issues.

## Introduction

Obesity is epidemic disease with excess energy storage and fat accumulation. An abnormally accumulated fat is positively associated with diabetes, cardiovascular diseases, hyperlipidemia, hypertension, metabolic syndrome, fatty liver disease, a certain type of cancer, depression, and anxiety (Fulton et al., 2022). The high prevalence of obesity is imposing an enormous burden on people’s health. A number of studies have shown the impact of obesity on infectious diseases such as coronavirus disease 2019 (COVID-19), it might play a role in the progression of COVID-19 after infection (Pan et al., 2021), and increased risk of hospitalization and severe illness from COVID-19 (Loos and Yeo, 2022). Over the past decades, medical treatment of obesity mainly focuses on the following mechanisms: 1) promoting energy expenditure; 2) lowering calorie absorption and 3) reducing energy intake. Pharmacological management of obesity has an overlong history populated with multiple prominent disappointments (Tak and Lee, 2021). Lifestyle management, lipid reduction, and weight loss surgery, which measures to reduce or absorb food intake, as well as increase its utilization are the main tools used to treat obesity today. However, body weight increases and recovery is associated with adverse drug reactions, surgery, and other problems, without effective and safe therapies for obesity (Ghasemi and Jeddi, 2017). Meanwhile, some Western medicines for obesity have reported side effects such as gastrointestinal reactions, cardiovascular complications, and negative moods. TCM has developed over a long history, it accumulated extensive pharmacological information and clinical experience to treat obesity. The effectiveness and safety of TCM offer an alternative therapy for this unsolved medical problem, e.g., traditional Chinese herbs and acupuncture (Sui et al., 2012). According to the TCM theory of Health in “Huangdi Neijing,” obesity has been indicated as spleen and kidney dysfunction, deficiencies, damp heat, and blood stasis. TCM treats obesity based on the restoration of an internal balance by eliminating dampness and heat, invigorating the kidney and spleen, activating blood circulation, and dispersing stagnated liver. The underlying mechanisms involved in reducing oxidative stress, anti-inflammation, and inhibiting lipid activity such as production and accumulation, which enhance leptin sensitivity and adiponectin (Cho et al., 2009).

Traditional Chinese medicine consists of complex ingredients from plants, minerals and animals. The traditional Chinese herbs were generally prescribed for treating illnesses according to the overall symptoms and collective experience, but this lacks the strict experimental proof to illustrate the features of clear-cut molecular compositions, pharmacokinetic profile, and mechanism of action. Addressing the tremendous challenge of some chemical complexity of TCM and overcoming bottlenecks in the drug development from herbal medicines requires the combination of modern technology, and scientific thought, as well as the traditional theory (Chu et al., 2020). Recently, many innovative technologies and methods are being accomplished in the research of TCM such as computer-aided drug design (CADD), cell membrane electrophysiology, gene hybridization, gene chip, luminescence, and fluorescent probe, DNA gel electrophoresis, and differential mRNA display technology (Li et al., 2008). Thus, a combination of Chinese medicinal herbs with modern scientific technology provides us to have wide insights and guides the development of promising powerful novel drugs. Several Chinese medicinal herbs are possible candidates for obesity, but their compositions and mechanisms are vague. It is important to incorporate herbal medicines into standard Western drug development pipelines and ultimately expand them as promising drugs or promising alternatives in the management of obesity. This article focuses on the concept of “Prodrug,” and describes a series of Chinese medicinal herbs, e.g., “Curcumin,” “Ginsenoside,” “Celastrol,” “Berberine,” “Artemisinin,” and “Capsaicin” as potential prodrugs for anti-obesity, and the combination of prodrug as well as the nanotechnology effect on obesity.

### Prodrug

Adrien Albert first announced the definition of “prodrug” in 1951 (Albert, 1958). The prodrug is inactive or less than the fully active form and converted into active compounds (drugs) through the metabolic pathways by enzymes such as hydrolases or other chemical actions within the human body. This undergoes a series of biochemical transformations to exert its pharmacological functions for targeting diseases (Cho and Yoon, 2018). In general, prodrugs are modified from parent molecule and selectively take effects in the target tissues, subsequently improving pharmaceutical (PH), pharmacokinetic (PK), and pharmacodynamics (PD) effects, as well as preventing undesirable side effects (Abet et al., 2017). The prodrug process more focuses on optimizing the ADMET (absorption, distribution, metabolism excretion, and toxicity) properties of potential herbal ingredients, and eventually increasing their efficiency and safety. These attach the poor water-soluble compound to a phosphate ester to increase its aqueous solubility (Raimund et al., 2011). In the presence of esterase, lipophilicity and membrane permeability of most prodrugs are increased, through esterase bioconversion, releasing rate of the prodrug is fostered (Perez et al., 2013).

Prodrugs exist in two types, carrier-bound and bioprecursors (Wermuth, 2008). Carrier-bound prodrugs consist of an active ingredient (drug) and a non-toxic carrier or promoiety *via* a covalent bond. The active ingredients are released from TCM through the action of enzymatic or non-enzymatic for covalent bond breaking (Shah et al., 2017). The carrier-linkers such as amides, esters, carbamates, phosphates, oximes, carbonates, N-Mannich, and imine are the main groups of prodrugs (Gandhi et al., 2019). Bioprecursors are the parent drug obtained by enzymatic transformation or biotransformation, *via* hydration, reduction, and oxidation (Shah et al., 2017).

**FIGURE 1:**
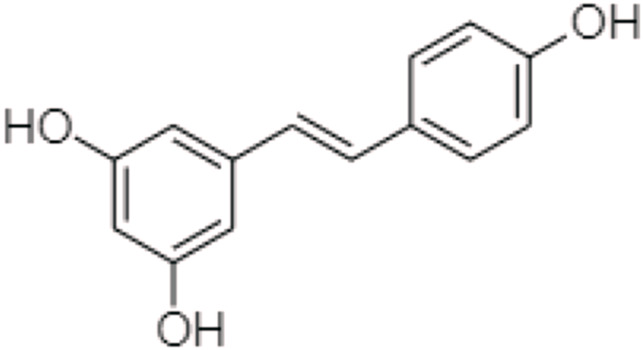
Chemical structure of Resveratrol.

**FIGURE 2:**
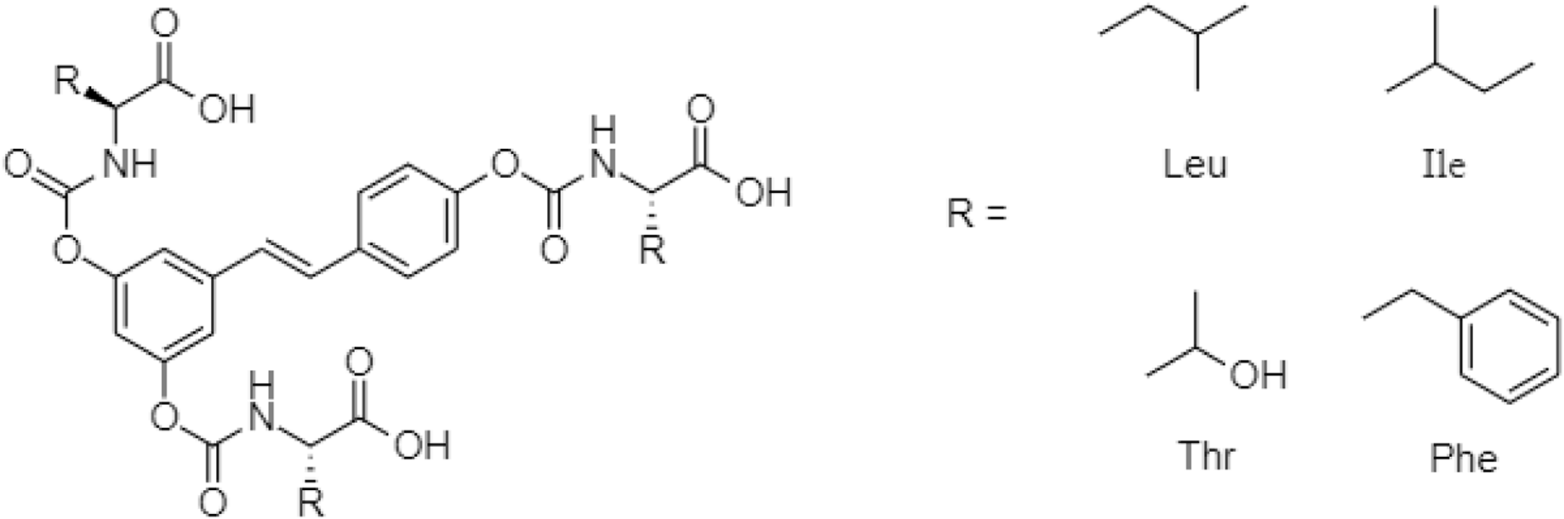
Chemical structures of amino acid substituted prodrugs.

One TCM prodrug example is “Resveratrol” for anti-obesity that can exist in the carrier-bound and bioprecursors, respectively. It is an active ingredient from TCM which is linked with the amino acid carbamate (Figure 1). The N-monosubstituted carbamate ester (-OC(O)NHR) as a carrier bound reacted to the OH group, preventing the binding of natural amino acids, e.g., Leu, Ile, Phe, Thr (Figure 2) and improving their molecular physicochemical properties, that significantly enhanced the absorption and stability of resveratrol in the bloodstream (Mattarei et al., 2015).

**FIGURE 3:**
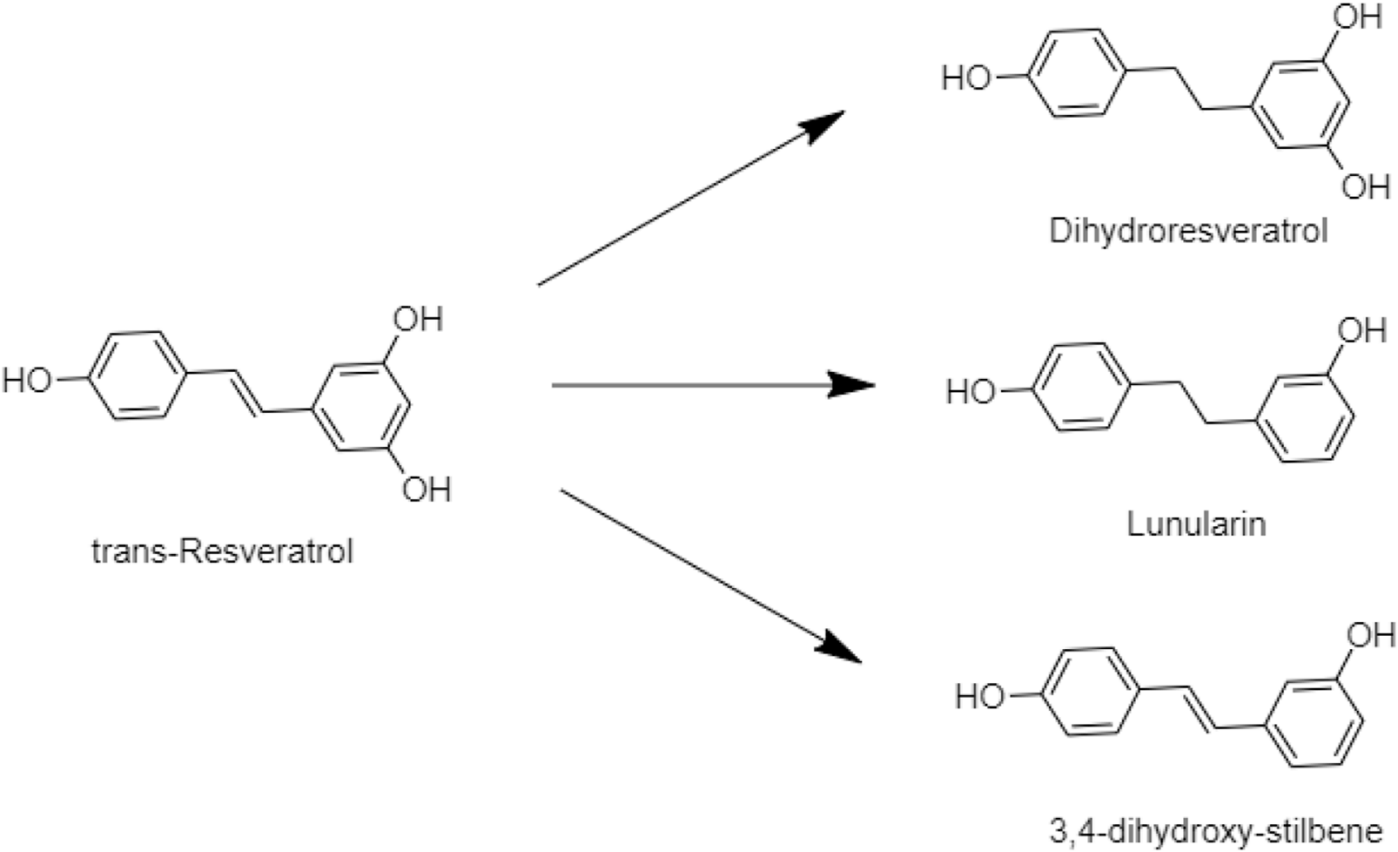
Example of gut microbiota biotransformation of trans-resveratrol.

Trans-Resveratrol is the isotope of resveratrol, which is formed *via* the reduction by the human gut bacteria. That improved the bioavailability of resveratrol *via* biotransformation and confirmed dihydroresveratrol, lunularin, and 3,4-dihydroxy-stulbene as prodrugs of resveratrol metabolism (Figure 3) (Kokil and Rewatkar, 2010; Bode et al., 2013; Zawilska et al., 2013).

Most Chinese herbal medicines are poor aqueous solubility, low bioavailability and stability, and non-target specificity, limiting their clinical applications. The above resveratrol prodrug examples indicated that prodrug improved solubility, absorption as well as stability of active ingredients. On the other hand, it might enable tissue-selective delivery and *in situ* activation (Testa, 2009). Thus, Chinese medicinal herbs are a promising source and suitable for the research and development of prodrugs to treat obesity or other metabolic diseases.

### Chinese medicinal herbs

Many traditional medicinal herbs and their ingredients have been tested and practiced over the years, such as Curcumin¸ Ginsenoside, Celastrol, Berberine, Artemisinin, and Capsaicin. These are natural Chinese herb-based therapeutics for obesity.

**FIGURE 4:**
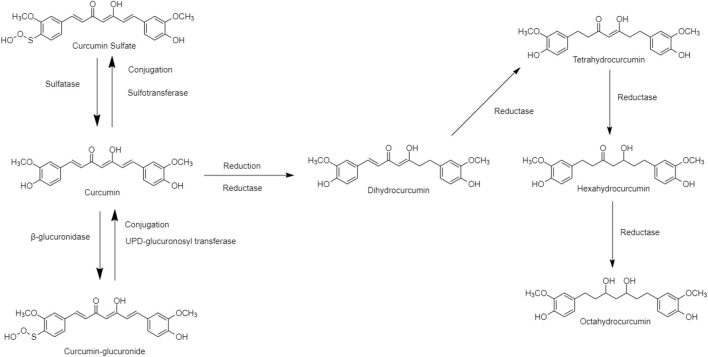
Chemical structures of curcumin and its derivative through the “reductase” metabolism.

Curcumin, an essential active ingredient in traditional Chinese turmeric (Curcuma longa), possessed multiple pharmacological effects including antioxidant, anti-inflammatory, antiviral, antimicrobial, and antitumor functions (Koboziev et al., 2020; Law et al., 2022; Nurcahyanti et al., 2022). The physiological and pharmacological properties depend on the curcumin metabolites, e.g., Dihydrocurcumin (DHC), Tetrahydrocurcumin (THC), Hexahydrocurcumin (HDC), and Octahydrocurcumin (OHC). These curcumin metabolites act as prodrugs to interact with the human metabolic pathways, existing in Phase I and Phase II. “Reductase” is an enzyme in the phase I human metabolic pathway for the reduction of double bonds from curcumin, which converts it into di, tetra, hexa, and octahydrocurcumin. “Glucuronide” or “Sulfate” is a carrier bound in phase II human metabolic pathway conjugated to curcumin producing hydrogenated metabolites (Figure 4) (Ireson et al., 2002). The structure of curcumin derivatives contains two phenolic hydroxyl groups, it is easy to form hydrogen or ionic bonds and dissociate into negatively charged phenolic ions in the water, altering some protein functions in the human body (Wink, 2015). Curcumin metabolites may suppress the NF-κB in adipose tissue, and regulate the level of TNF-a, IL-6, monocyte chemotactic protein (MCP-1), plasminogen activator inhibitor type-1 (PAI-1), and increasing adiponectin expression to ameliorate the obesity risk factors (Bradford, 2013; Soleimani et al., 2018; Wink, 2022).

Obesity has corresponded to insulin resistance and diabetes (Bastard et al., 2006). NF-E2-related factor 2 (NRF2)-Kelch-like ECH-associated protein 1 (KEAP1) pathway and endoplasmic reticulum (ER) stress are the two important factors in the primary adipocytes causing the risk of obesity (Wang et al., 2016); while curcumin and its metabolites are the signaling modulators to improve the lipolysis and insulin resistance for preventing obesity (Ye et al., 2017).

Sterol regulatory element-binding protein 1C (SREBP-1C), peroxisome proliferator-activated receptor gamma (PPARγ), fatty acid synthase (FAS), and fatty acid-binding protein 4 (FABP4) are the lipogenic proteins that inhibited by the THC. It ameliorates free fatty acid-induced hepatic steatosis and regulates the secretion of insulin, as well as significantly decreased lipid accumulation that leads to non-alcoholic fatty liver disease (NAFLD). As the phosphorylation of an insulin receptor substrate 1 (IRS-1)/phosphoinositide 3-kinase (PI3K)/Akt and downstream signaling pathways, forkhead box protein O1 (FOXO1), and glycogen synthase kinase 3β (GSK3β) are modulated by THC to decrease the risk of obesity (Chen et al., 2018).

DHC manages glucose uptake, reduces the risk of obesity, and prevents diabetes because it regulates the levels of sterol regulatory element binding protein-1C (SREBP-1C), patatin-like phospholipase domain containing 3 (PNPLA3), and peroxisome proliferator-activated receptor-α (PPARα) in the mRNA and proteins It decreases the level of triglycerides (TG) when the phosphatidylinositol-3-kinase (PI3K) and phosphorylated serine-threonine protein kinase (pAKT) increases to improve lipid accumulation, oxidative stress, and insulin resistance (Yu et al., 2018).

Besides, DHC and THC can alleviate adiposity and suppress inflammatory responses as well as improve insulin sensitivity in white adipose tissue, which are regulated through the FNDC5/p38 mitogen-activated protein kinase (p38 MAPK) or extracellular signaling-related kinase (ERK) signaling pathways. These increase oxygen consumption and thermogenesis, also the respiratory exchange ratio within the body for reducing the risk of obesity (Zou et al., 2021).

The THC is also a potential agent to attenuate in a fatty food or streptozotocin causing adiposity, steatosis, and hyperglycemia. It is a novel therapeutic use for type 2 diabetes since it is improving insulin signaling, glucose utilization, and lipid metabolism through the AdipoR1/R2-APPL1-mediated pathway, which upregulated *via* the uncoupling protein 1 (UCP-1) in adipose tissue and elevated adiponectin levels in the circulation of liver and skeletal muscle (Tsai et al., 2021).

**FIGURE 5:**
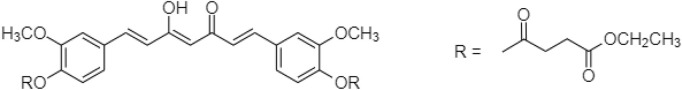
Chemical structure of curcumin diethyl disuccinate (CurDD).

In 2019, Muangnoi et al. (2019) reported a prodrug for curcumin linked with diethyl succinate ester. “Curcumin diethyl succinate” (Figure 5) enhanced curcumin’s anti-proliferative effect on HepG2 cells *via* apoptotic induction. Recent study showed caspase induction and Bcl-2 inhibition for altering insulin signaling in human adipose tissue to prevent obesity.

**FIGURE 6:**
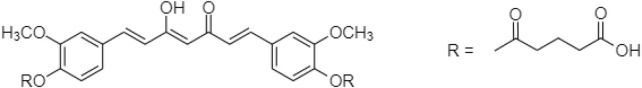
Chemical structure of curcumin diglutaric acid.

Phumsuay et al. (2020) also identified another curcumin ester prodrug, which was linked with diglutaric acid. The “Curcumin diglutaric acid” (Figure 6) was an anti-inflammatory agent for the edema model. The levels of pro-inflammatory cytokine expression such as NO, IL-6, TNF-α, iNOS, and COX-2 expression were reduced. Curcumin diglutaric acid was a pro- and anti-inflammatory mediator secreted by adipose tissue subsequently lower systemic inflammation and the risk of metabolic diseases *via* the suppression of MAPK (ERK1/2, JNK, and p38) activity (Jayarathne et al., 2017; Phumsuay et al., 2020).

**FIGURE 7:**
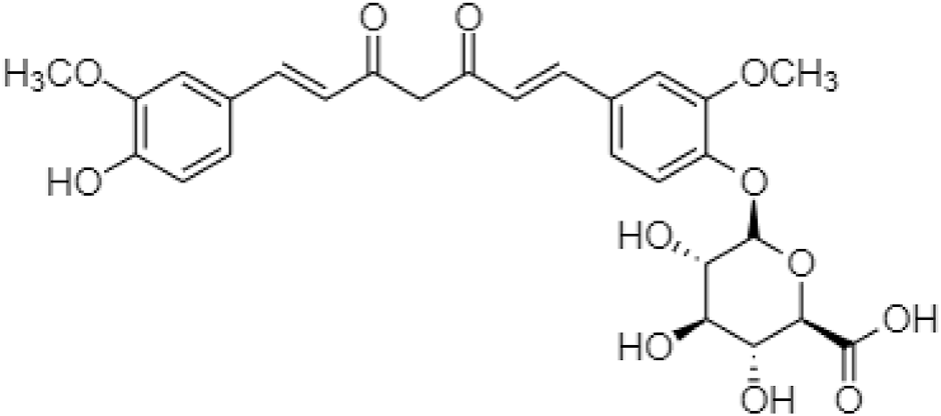
Chemical structure of curcumin D-gluronide.

Khadka et al. (2021) also synthesized a prodrug of curcumin with monoglucuronide (CMG) (Figure 7), altering the gut microbiota and immune responses to treat obesity. As low fecal microbial diversity is associated with increased total fat and dyslipidemia, higher low-grade inflammation, and homeostasis, thus CMG administration could affect both the overall gut microbiome compositions and the abundance of individual bacteria in feces, ileal contents, and the ileal mucosa (Davis, 2016; Yoo et al., 2021).

**FIGURE 8:**
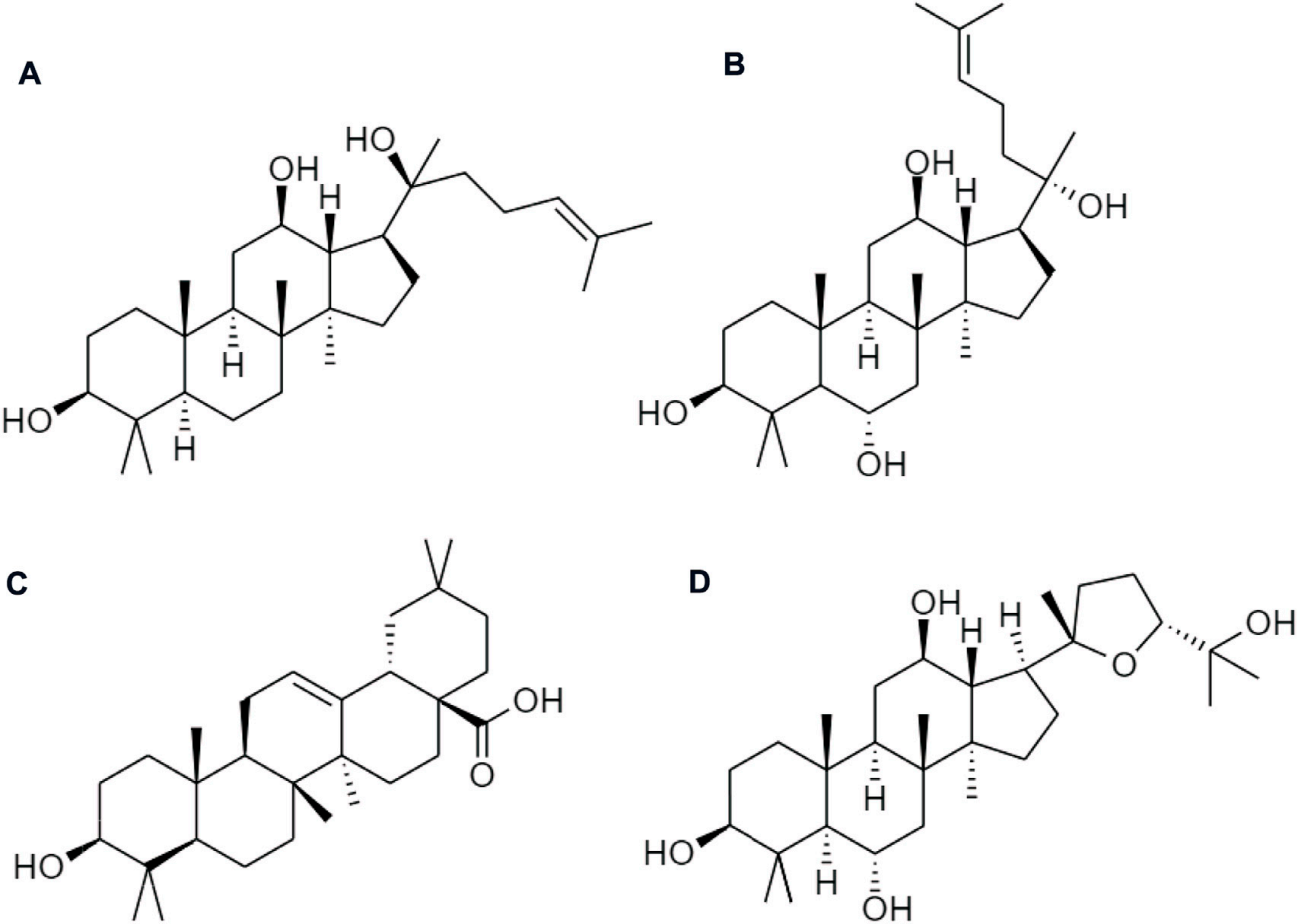
Chemical structures of **(A)** protopanaxadiol (PPD), **(B)** protopanaxatriol (PPT), **(C)** oleanane group (Ro), and **(D)** ocotillol type pseudoginsenoside.

Ginseng (Panax ginseng) has been widely used to improve human health. Asian countries are the most common but it is also used in the United States and Europe. Ginsenosides are the active ingredients, which involve a series of tetracyclic triterpenoid saponins. They are classified into oleanane type and dammarane type (17 carbons in a four-ring structure) according to their structural difference. Protopanaxadiol (PPD), and protopanaxatriol (PPT) groups are the prodrugs of ginsenosides from dammarane type. Similarly, an oleanane (Ro) type is sorted in the oleanolic acid group and ocotillol type pseudoginsenoside (Figure 8). Ra1, Ra2, Rb1, Rb2, Rc, Rd, Rg3, Rk1, Rg5, and Rh2, are the examples of PPD-type ginsenosides, *etc.*, compound K (CK), and PPD, whereas PPT-type ginsenosides consist of Re, Rg1, Rg2, Rf, Rh1, and PPT, *etc.* (Shi et al., 2019). Among those chemical ingredients, the contents of Rb1, Rb2, Rc, Rd, Re, Rf, and Rg1 contribute more than 90% to ginsenosides (Fan et al., 2020).

Ginsenosides possess anti-inflammatory, anti-stress, anticancer, anti-oxidative, and anti-aging activities for preventing obesity, hyperlipidemia, hyperglycemia, and hepatic steatosis (Yan et al., 2018). Ginsenoside Rb2 is a novel AMP-activated protein kinase (AMPK) activator that reduces body weight, improves insulin sensitivity, and induces energy expenditure in diet-induced obese (DIO) rats. As ginsenosides target the central nervous system (CNS), which regulates leptin sensitivity in the cerebral cortex to prevent obesity and decreases the risk of central inflammation in the hypothalamus (Jeon et al., 2021).

Ginsenosides are distinctive triterpenoid dammarane saponins with very low solubility for oral administration of their slow absorption, extensive microbial deglycosylation, biliary excretion, and degradation of acidic ingredients such as Rg1 and Rb1 (Liu et al., 2009; Liu et al., 2010). Almost all ginsenoside dammarane is instability in the gastrointestinal tract. That is readily degraded or metabolized to secondary glycosides, aglycones, and other metabolites by gastric acid and/or the intestinal microflora. The bioavailability of ginsenosides are improved to improve the absorption by increasing water solubility, biofilm permeability, and stability in the gastrointestinal tract. The larger molecular size of ginsenosides with lower membrane permeability of the dammarane skeleton, and the sugar fractions have hydrophilic properties that promote the dissolution of ginsenosides. However, many ginsenosides have been reported as aglycones and lack sugars, thus showing low water solubility. On the other hand, ginsenoside is readily degraded by gastric acid hydrolysis *via* C-20 sugar radical epimerization and glycosyl elimination (Yang et al., 2011; Cheung and Law, 2022).

**FIGURE 9:**
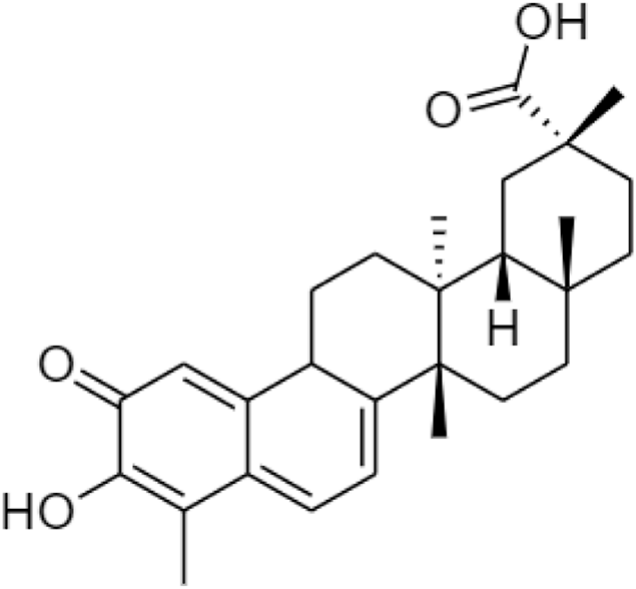
Chemical structure of celastrol.

Celastrol (Figure 9) belongs to the “Celastraceae” family. It is extracted from the root of *Tripterygium wilfordii* (Venkatesha and Moudgil, 2016) containing natural triterpene with anti-inflammatory, antioxidant, and anti-cancer activities (Straub, 2017). Since obesity and diabetes are also associated with an excessive inflammatory response (Liu et al., 2015), celastrol acts as a leptin sensitizer in the pharmacological treatment of obesity. It could block food intake, reduce energy expenditure, and lead to a weight gain of about 45% in hyperleptinemia diet-induced (DIO) obese mice. It also regulates the leptin sensitivity in either leptin-deficient (ob/ob) or leptin receptor-deficient (db/db) mouse models (Li et al., 2018).

**FIGURE 10:**
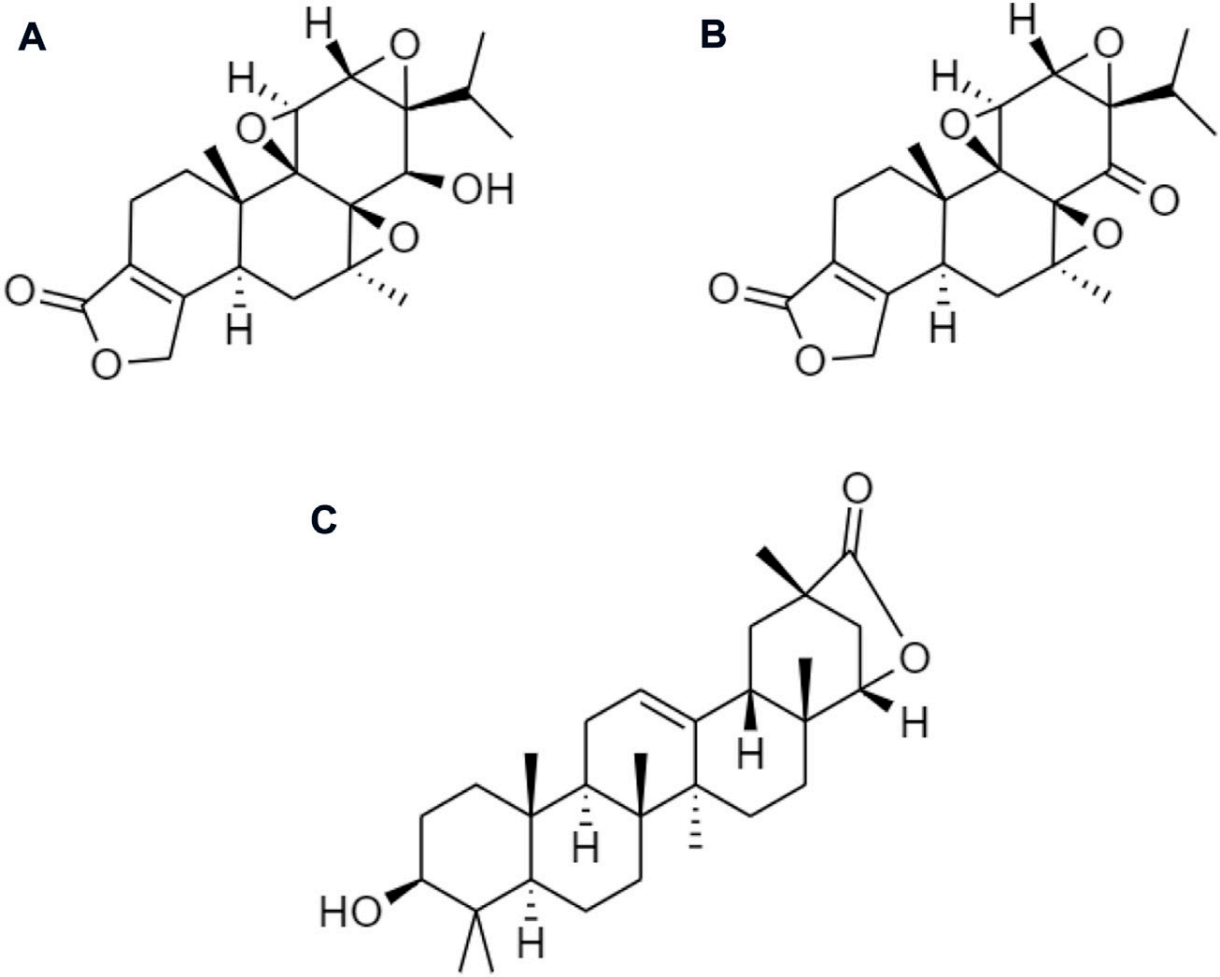
Chemical structures of **(A)** triptolide, **(B)** triptonide, and **(C)** wilforlide.

Other derivatives of celastrol such as **(A)** triptolide, **(B)** triptonide, and **(C)** wilforlide are also used as prodrugs for obesity (Figure 10) (Venkatesha and Moudgil, 2016). **(A)** Triptolide is an inflammatory mediator that inhibits the activation of the AMPK/mTOR signaling pathway to reduce the secretion of chemokine, monocyte chemotactic protein-1 (MCP-1) in Kupffer cells (KCs), Ma-CM, or 3T3-L1 to ameliorate obesity-induced inflammatory diseases (Li and Liu, 2021). **(B)** Triptonide without any toxic effect, but it may suppress the level of astrocyte-elevated gene-1 (AEG-1) (Fu et al., 2020). This regulates nuclear receptors to control lipid metabolism and reduces the risk of high-fat as well as high-cholesterol diets for obesity (Robertson et al., 2015). **(C)** Wilforlide improves rheumatoid arthritis progression, which inhibits M1 macrophage polarity and blocks Toll-like receptor 4 (TLR4) through activation of the NF-κB p65 pathway. It also deactivated the TLR4/NF-κB partially mediated signaling pathway (Cao et al., 2022) and modulates the obesity-induced inflammatory response (Rogero and Calder, 2018).

**FIGURE 11:**
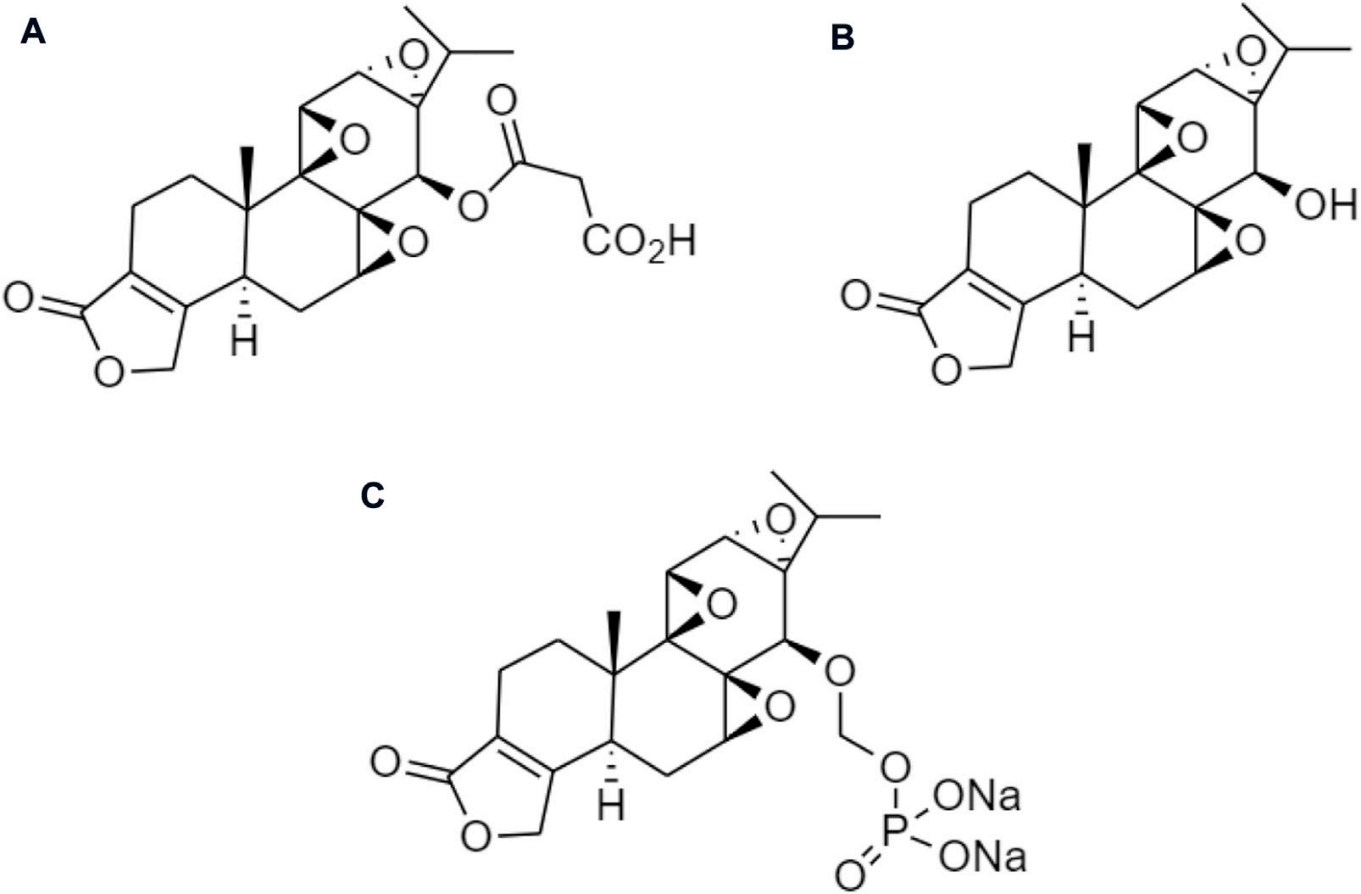
Chemical structures of **(A)** omtriptolide, **(B)** 5-hydroxytriptolide, and **(C)** disodium phosphonooxymethyl.

Several prodrugs of triptolide have been reported including omtriptolide, 5-hydroxytriptolide, and a disodium phosphonooxymethyl (Figure 11). These are involving carboxylic acid, amino acid esters, and a non-covalent interaction for targeting the protein to suppress kinases, and Hsp70 expression on the prevention of non-alcoholic fatty liver disease (NAFLD) progression and disodium phosphonooxymethyl reduce the activity of anti-inflammatory for obesity (Di Naso et al., 2015; Patil et al., 2015).

**FIGURE 12:**
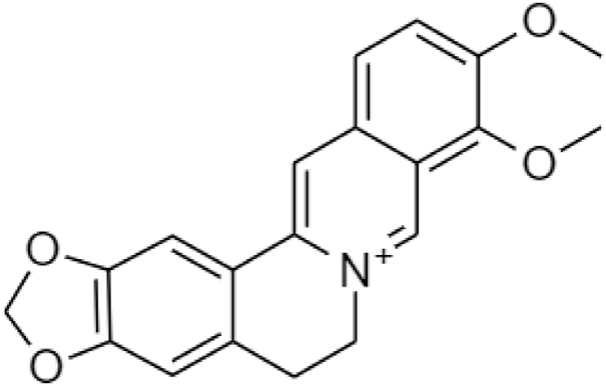
Chemical structure of berberine.

Berberine (Figure 12), is the major active ingredient from Coptis Chinensi. Berberine-containing medicinal plants have been applied in TCM to treat parasitic intestinal infection, bacterial diarrhea, and diabetes for a long time through the suppression of fatty acid synthase, acyl-CoA synthase, acetyl-CoA carboxylase, SREBP-1, C/EBPα, and PPARγ (Choi et al., 2006; Hu and Davies, 2009). Berberine blocks food absorption, reduces body gain, and visceral adipose weight by decreasing the level of PPARγ in high-fat diet-induced obesity mice when the level of GATA-binding proteins 3 increases and downregulates the expression of PPARγ and PPARα. GATA-binding proteins 2 and 3 (GATA-2 and GATA-3) are modulated differentiation of pre-adipocytes into mature adipocytes (Hu and Davies, 2010). Besides, gut bacteria act an important role to regulate the fat accumulation and its degradation metabolism, since berberine has antibacterial or anti-obesity activity. Han et al. (2011) reported that berberine lowered blood lipids and controls glycemic levels including free fatty acids and cholesterol, which activated thermogenesis in adipocytes and increase energy expenditure.

**FIGURE 13:**
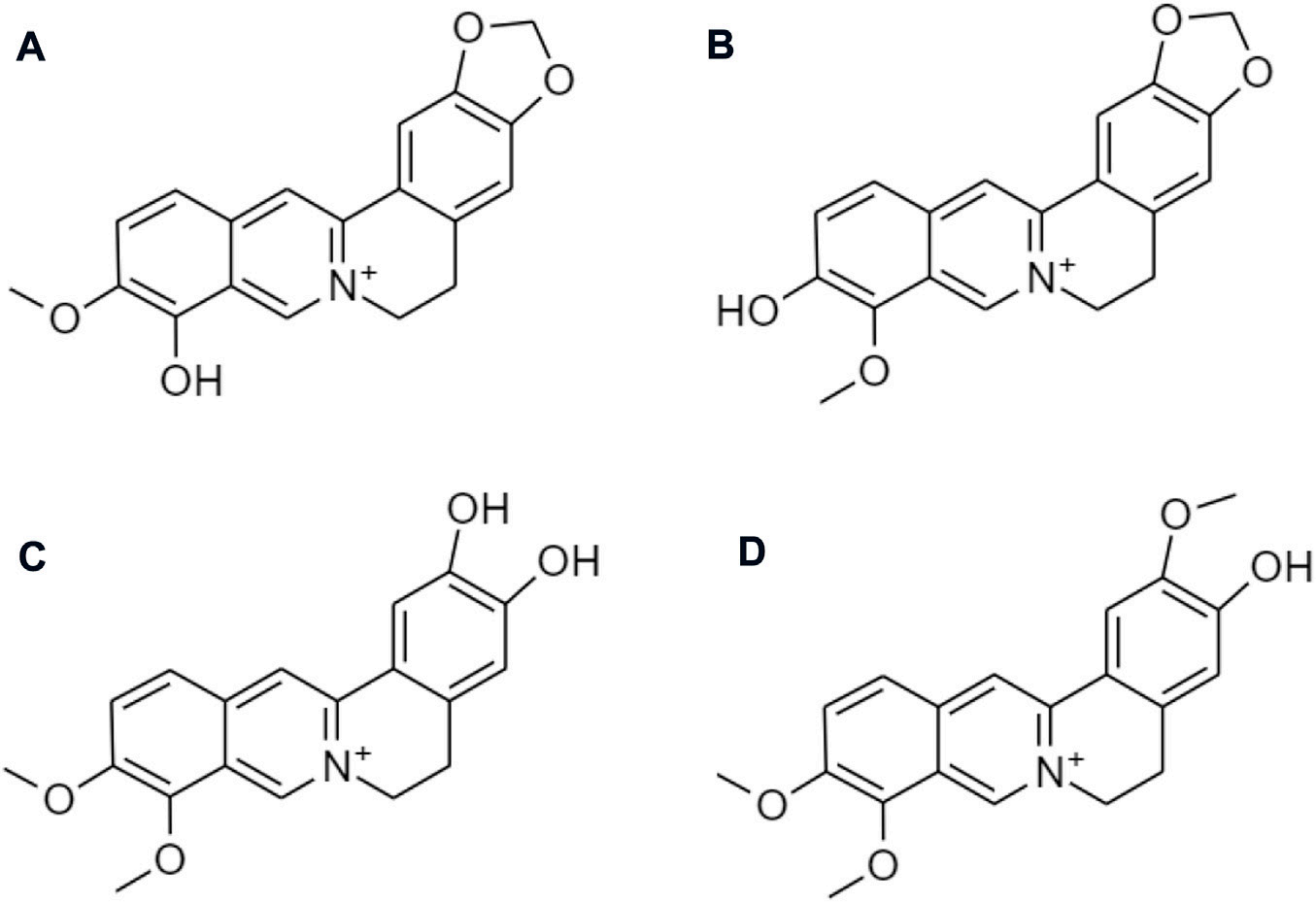
Chemical structures of **(A)** berberrubine, **(B)** thalifendine, **(C)** demethyleneberberine, and **(D)** jatrorrhizine.

Despite the various therapeutic effects, berberine has not been yet developed as a marketed drug product. Poor absorption, fast metabolism, and wide tissue distribution lead to very low bioavailability and limit its translation to clinical settings. Most of the berberine is absorbed in the small intestine *via* oral administration. There are several reasons which are responsible for its low absorption and bioavailability. The chemical structure of berberine is a hydrophilic compound, which makes it difficult for it to cross the plasma membrane of intestinal cells. An intestinal first-pass elimination is extensive, around 50% of the berberine keep intact through the gastrointestinal tract. Berberrubine, thalifendine, demethyleneberberine, and jatrorrhizine are the four major metabolites (Figure 13) after being metabolized by the CYP450 isoenzyme (Li et al., 2011).

Meanwhile, berberine is the substrate, which is regurgitated into the intestinal lumen by ATP-binding cassette (ABC) transporters. The ABC transporters include multidrug resistance-associated protein (MRP) and P-glycoprotein [P-gp, also named multidrug resistance protein1 (MDR1)] (Shitan et al., 2003).

**FIGURE 14:**
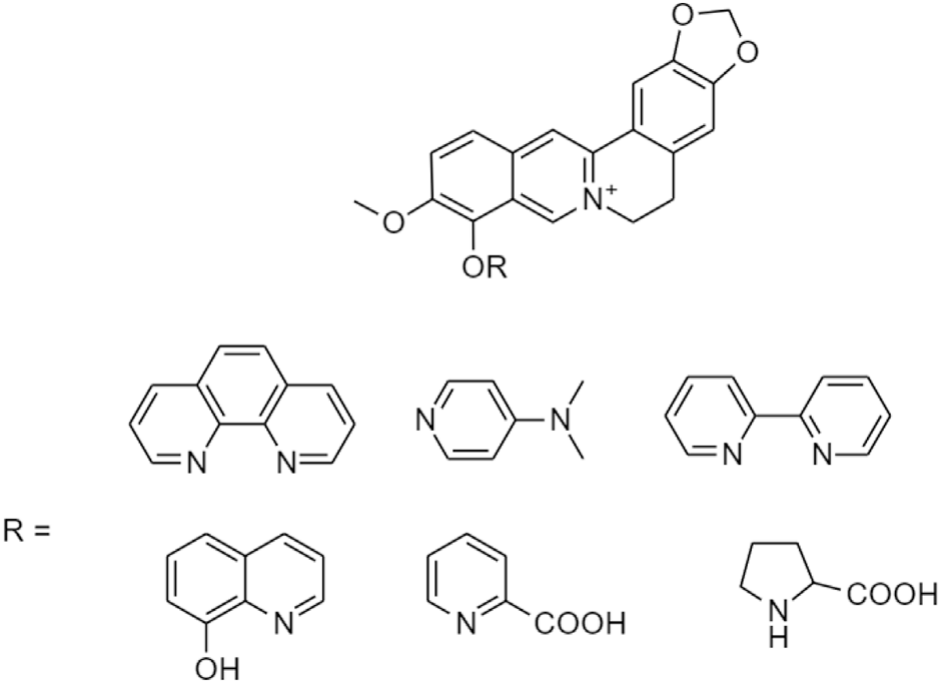
Chemical structures of 9-O-Aryl berberine and its derivatives.

In 2020, Habtemariam (2020) discovered a prodrug of berberine, 9O-Aryl berberine (Figure 14) for type-2 diabetes and associated diseases. It suppresses gluconeogenesis and lipid accumulation to prevent obesity through the inhibition of hepatocyte nuclear factor-4α (HNF-4α) and the microRNA miR122.

**FIGURE 15:**
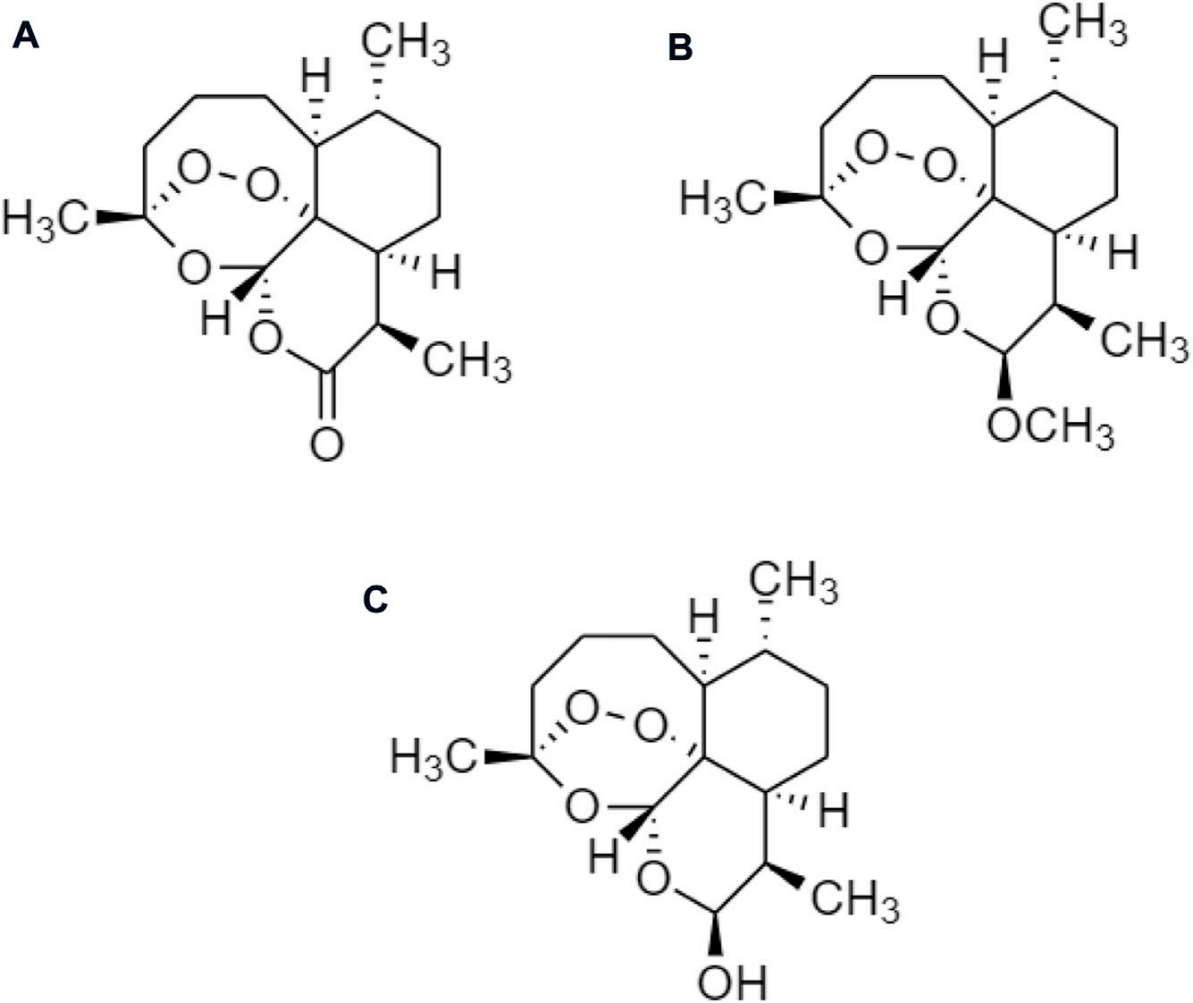
Chemical structures of **(A)** artemisinin, **(B)** artemether, and **(C)** dihydroartemisinin.

Artemisinin is derived from *Artemisia annua* (qinghao). Artemether and dihydroartemisinin (Figure 15) are the derivatives with anti-inflammatory properties, suitable for the inflammation of chronic metabolism (Law et al., 2020), which are implicated in their pathogenesis corresponding to obesity or other metabolic disorders (Shen et al., 2020). The artemisinin derivatives induce the accumulation of the browning of white adipose tissue (WAT) within the body and also enhance the brown adipose tissue (BAT) function to reduce the risk of obesity. PRDM16, PGC1a, and UCP1 are the brown genes upregulated by artemether and dihydroartemisinin, through the activation of the p38 MAPK/ATF2 axis and the deactivation of Akt/mTOR pathway for browning of C3H10T1/2 cells (Lu et al., 2016).

**FIGURE 16:**
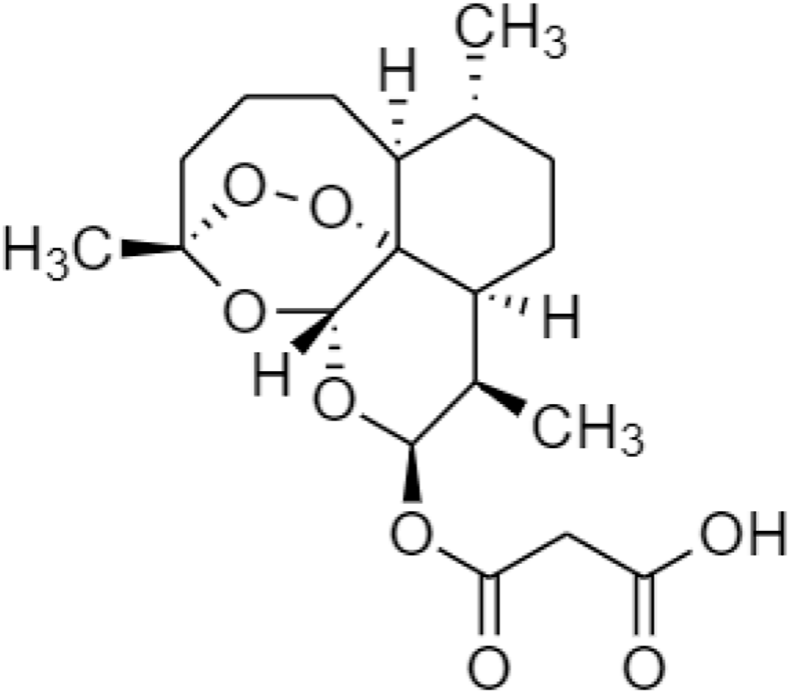
Chemical structure of artesunate.

In 2007, Chung MC *et al.* reported a prodrug of dihydroartemisinin, “artesunate” through reduction and esterification using diisobutylaluminum hydride (DIBAL) and succinic anhydride (Figure 16). The prodrug with low toxicity and substantially increased solubility in water (Chung et al., 2007). The mechanism of artesunate for obesity included blocking the NF-κB pathway, inhibiting iNOS expression, and decreasing NO production (Jiang et al., 2020).

**FIGURE 17:**
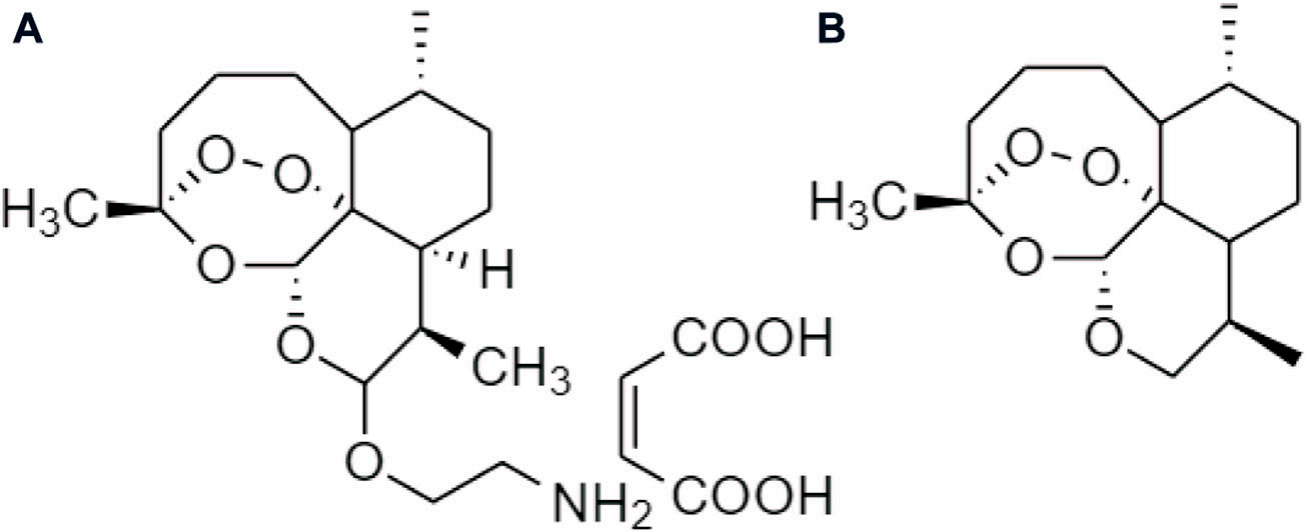
Chemical structures of **(A)** SM934 and **(B)** ADRT.

Zhang Y *et al.* also developed some prodrugs of artemisinin, such as SM934 and Anhydrodehydroartemisin (ADRT) (Figure 17). The SM934 ameliorated the experimental autoimmune encephalomyelitis (EAE) relating to obesity (Zhang et al., 2022a); whilst ADRT reduced the functions of CNS and decreased the immunity of a peripheral system (Lv et al., 2021). It inhibits adipogenesis and the implications for obesity, as well as inflammation due to the induction of a pro-inflammatory cytokine interleukin IL-17A, Th1/Th17, and IFN-γ (Ahmed and Gaffen, 2010).

**FIGURE 18:**
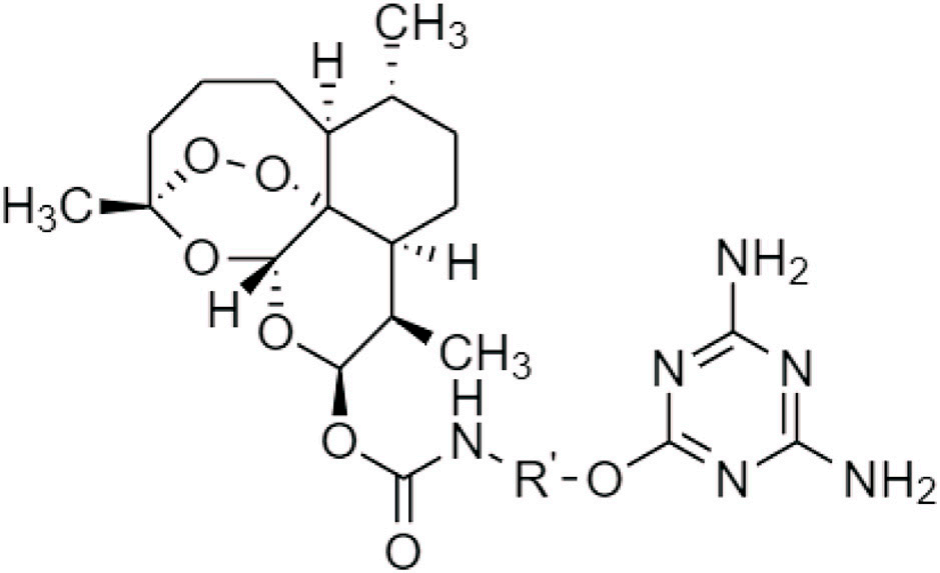
Chemical structure of artemisinin linked with hydrocarbylene.

Additionally, an artemisinin prodrug *via* modification of hydrocarbylene (Figure 18) with long-chain hydrocarbons was reported. It was a lipase inhibitor in the stomach and pancreas for reducing the absorption of dietary fats to manage obesity (Heck et al., 2000).

**FIGURE 19:**
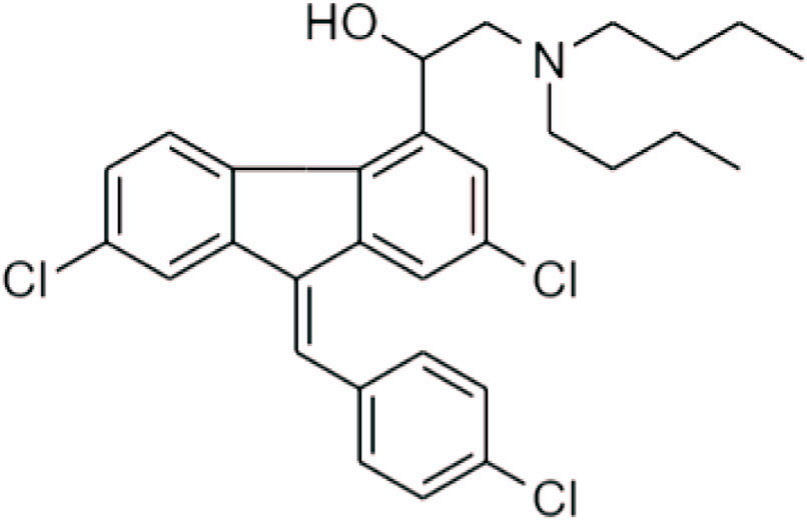
Chemical structure of artemether-lumefantrine.

Recently, Sugiarto SR *et al.* investigated the pharmacokinetic properties of other prodrugs in artemisinin, which was the combination of artemether-lumefantrine (Figure 19) therapy for normal-weight, overweight, and obese male adults. Artemether and lumefantrine were metabolized through the hepatic CYP450 enzyme to produce protease inhibitors (PIs), and non-nucleoside reverse transcriptase inhibitors (NNRTIs), which suppressed the concentration of plasma to treat overweight (Sugiarto et al., 2022).

**FIGURE 20:**
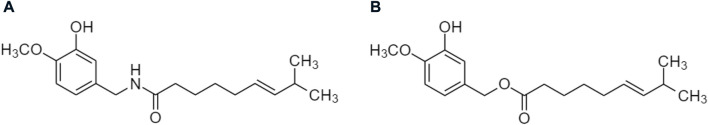
Chemical structures of **(A)** capsaicin and **(B)** capsaicinoids.

Chili pepper belongs to the genus Capsicum, which is an important spice widely consumed in various diets. It is used to treat pains, asthma, coughs, sore throat, common cold, and arthritis in TCM. Its unique components, including capsaicin and capsaicinoids (Figure 20), contribute to the pungent scent and heat sensation of hot chili pepper (Azlan et al., 2022).

Capsaicin (CAP) has many pharmacological benefits to humans, such as pain-alleviating, anti-inflammation, antioxidant, hypoglycemic, anticancer, antimicrobial, and anti-obesity effects. This is a potential agent for obesity by inhibiting fat accumulation and lipid oxidation, increasing the satiety of the hypothalamus, and preventing appetite and fat consumption. Glycerol-3-phosphate dehydrogenase (GDPH) and intracellular triglyceride are decreased in 3T3-L1 adipocytes because CAP inhibits the level of PPAR, C/EBP, and leptin. Besides, capsaicin significantly lowers the serum level of glucose and lipid including cholesterol and triglycerides in mice (Mahalak et al., 2022). Wang *et al.* proposed that capsaicin could change the structure and composition of gut microbiota to lower lipid absorption (Wang et al., 2020).

**FIGURE 21:**
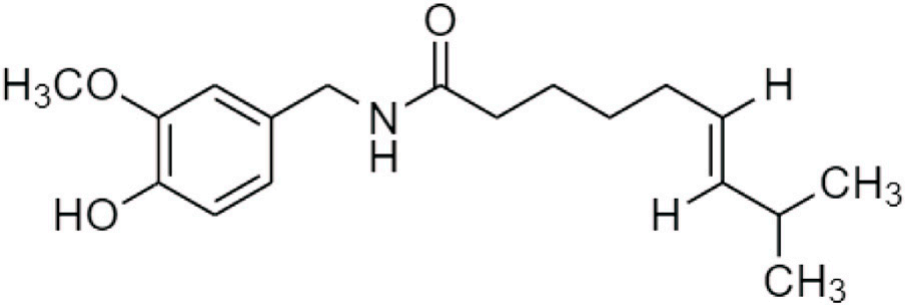
Chemical structure of trans-CAP, vocacapsaicin (trans-8-methyl-N-vanillyl-6-nonenamide).

In 2021, Higgins et al. (2021) reported a prodrug of trans-form CAP, vocacapsaicin (trans-8-methyl-N-vanillyl-6-nonenamide) (Figure 21). This was a phenolic compound responsible for their characteristic taste and pungency. It was a water-soluble compound that rapidly converted to capsaicin. The transient receptor potential vanilloid 1 (TRPV1)-agonist was applied in the trigeminovascular system. CAP modulated the fasting blood glucose and insulin concentrations as well as decreased the levels of proinflammatory cytokines interleukin-1β and interleukin-6 to treat high-fat, or high-sucrose (HFHS) diet-induced obesity (Marics et al., 2017).

However, trans-CAP with high concentration inhibits gastric acid production, causing gastric inflammation and damaging our gastrointestinal mucosa. As a potent irritant, CAP with severe irritation, pain, and burning if exposed to the mucous membranes. Capsaicin with low bioavailability and a short half-life. It is rapidly metabolized in all tissues including the liver, kidney, intestine, serum, and blood after oral administration (Knotts et al., 2022).

## Discussion

The prodrug is widely used in drug development because it is bio-reversible, inactive drug derivatives, and non-toxic, which can be converted into an active drug within the human body (Markovic et al., 2020). Prodrugs have many potential benefits with patient compliance, and more efficient absorption, distribution, metabolism, and excretion (ADME) properties that are suitable for lots of traditional Chinese medicines (TCMs). Some active compounds of TCM are insoluble and have lower absorption for the human body. Prodrugs of TCM improve the stability, increase solubility, and bioavailability of traditional Chinese medicines and reduce their toxicity, as well as foster the targetability.

Currently, prodrugs are further developed through the combination of “Nanotechnology.” Prodrugs with nanotechnology have many advantages, e.g., greater bioavailability, higher drug encapsulation, and loading efficiency, as well as the control of drug release (Luo et al., 2014; Lang et al., 2021; Zhang et al., 2021; Zhang et al., 2022b). Growing evidence has shown that the combination of nanotechnology and prodrug is more effective to treat obesity than the usage of prodrugs alone. In general, prodrugs processing with nanotechnology increase the drug solubility. It might fasten or increase the absorption rate of the original drug in an organ, which targeted the drug delivery to enhance the treatment effective rate and decrease the side effects, as well as the drug dose (Bahadori et al., 2019). These have been illustrated with the following examples:

(i) Ahmed et al. (2021) reported that curcumin-loaded chitosan/polyethylene glycol (PEG) blended poly lactic-co-glycolic acid (PLGA) nanoparticles, which reduced the body weight, body mass index (BMI), and Lee index. This diminished the weights of the liver, heart, and visceral adipose tissues. It also significantly reduced the weights of the gonadal and subcutaneous adipose tissues. (ii) Kim et al. (2018) discovered a diverse micro-/nano-sized delivery system using emulsions, polymers, and vesicles for improving bioavailability and maximizing the therapeutic potential of ginsenosides. The ginsenoside was easily absorbed by the human body as it was converted into secondary saponins and aglycones *via* this nano-system (Kim et al., 2018; Li et al., 2022), decreased body weight, regulated the concentration of insulin, and reduced energy expenditure to change the obese situations.
(iii) Liu et al. (2012) identified that celastrol linked with the chitosan, O-carboxymethyl chitosan, or N-[(2-hydroxy-3-N,N-dimethylhexadecyl ammonium)propyl] chitosan chloride was effective to treat overweight and insulin resistance in obesity, which decreased body mass, regulated the plasma glucose, insulin and leptin sensitivity, as well as an increased fecal lipid for preventing obesity.
(iv) Och et al. (2020) applied lysergol, D-α-tocopheryl polyethylene glycol 1000 succinate (TPGS), chitosan, and sodium caprate as polymers to form an anhydrous reverse micelle (ARM) delivery system of berberine nanoparticles for enhancing the oral bioavailability and absorption. The formulations of berberine increased the bioavailability of berberine compared to berberine alone. Berberine nanoparticle was an anti-obesity and anti-sclerotic agent by lowering the low-density lipoprotein (LDL) and testosterone levels. It stimulated glycolysis, improved insulin secretion, and inhibited gluconeogenesis and adipogenesis to treat obesity.
(v) Du et al. (2022) introduced a liposome-like nanosystem in the self-assembly of dimeric artesunate glycerophosphocholine conjugated to the antimalarial drug dihydroartemisinin (DHA) for killing parasites. Similarly, this liposome-like nanosystem of artemisinin might induce the differentiation and decrease of regulatory cells to minimize the number of adipose cells in obesity.
(vi) Lu et al. (2017) developed capsaicin-loaded nanoemulsions (C-NE) for enhancing the anti-obesity effect on gastric mucosa to degrade the high-fat diet. It also alleviated stomach inflammation to keep gastrointestinal smoothness prevent the adipose cells’ accumulation and reduce the risk of obesity.

## Conclusion

Obesity is a global problem and promotes the incidence of various diseases, particularly cardiovascular disease, metabolic syndrome, diabetes, hypertension, osteoarthritis, and certain cancers. The Chinese herbal medicines’ derivatives, such as Curcumin¸ Ginsenoside, Celastrol, Berberine, Artemisinin, and Capsaicin are suitable Chinese medicinal herb-based therapeutics for Obesity. The above derivative as prodrugs are the possible approach to improve their poor solubility, absorption, and bioavailability. Up to the present, a combination of prodrugs from Chinese medicinal herbs and nanotechnology is the innovative strategy for treating obesity. However, much more works need to be done, especially in the clinical trial.

## Glossary

**ADME:**: Absorption, Distribution, Metabolism, Excretion
**ADRT:**: Anhydrodehydroartemisin
**AEG-1:**: Astrocyte Elevated Gene-1
**AMPK:**: AMP-activated protein kinase
**ARM:**: Anhydrous reverse micelle
**BAT:**: Brown adipose tissue
**CADD:**: Computer-aided drug design
**CMG:**: Curcumin bound with monoglucuronide
**C-NE:**: Capsaicin-loaded nanoemulsions
**CNS:**: Central nervous system
**DHC:**: Dihydrocurcumin
**DIO:**: Diet-induced obese
**EAE:**: Experimental autoimmune encephalomyelitis
**Eotaxin:**: Eotaxin-1/Eotaxin-2/Eotaxin-3
**ER:**: Endoplasmic reticulum
**ERK:**: Extracellular signal-related kinase (ERK)
**FABP4:**: Fatty acid-binding protein 4
**FAS:**: Fatty acid synthase
**FOXO1:**: Forkhead box protein O1
**GATA-2 & GATA-3:**: GATA binding protein 2 and 3
**GDPH:**: Glycerol-3-phosphate dehydrogenase
**GSK3β:**: Glycogen synthase kinase 3β
**HDC:**: Hexahydrocurcumin
**HNF-4α:**: Hepatocyte nuclear factor-4α
**HSP90:**: Heat shock protein 90
**IRS-1:**: Insulin receptor substrate 1
**KC:**: Kupffer cells
**LDL:**: Low-density lipoprotein
**LPS:**: Lipopolysaccharides
**MAPK:**: Mitogen-activated protein kinase
**MCP-1:**: Monocyte Chemoattractant Protein-1
**MS: Multiple sclerosis**
**NAFLD:**: Non-alcoholic fatty liver disease
**NF-κB:**: Nuclear factor kappa-light-chain-enhancer of activated B cells
**NNRTIs:**: Non-nucleoside reverse transcriptase inhibitors
**NRF2-KEAP1:**: NF-E2-related factor 2-Kelch-like ECH-associated protein 1
**OHC:**: Octahydrocurcumin
**PAI-1:**: Plasminogen activator inhibitor-1
**pAKT:**: Phosphorylated serine-threonine protein kinase
**PD:**: Pharmacodynamics
PEG: Polyethylene glycol
**PH:**: Pharmaceutical
**PI3K:**: Phosphatidylinositol 3-kinase
**PIs:**: Protease inhibitors
**PK:**: Pharmacokinetic
**abrPLGA:**: Poly lactic-co-glycolic acid
**PNPLA3:**: Patatin-like phospholipase domain containing 3
**PPARα:**: Peroxisome proliferator-activated receptor-α
**PPARγ:**: Peroxisome proliferator-activated receptor gamma
**PPD:**: Protopanaxadiol
**PPT:**: Protopanaxatriol
**Rantes:**: Regulated on activation, normal T cell expressed and secreted
**ROS:**: Reactive oxygen species
**SREBP-1C:**: Sterol regulatory element-binding protein 1C
**TCM:**: Traditional Chinese medicine
**THC:**: Tetrahydrocurcumin
**TLR4:**: Toll-like receptor 4 Beclin-1
**TPGS:**: D-α-tocopheryl polyethylene glycol 1000 succinate
**TRPV1:**: Transient receptor potential vanilloid 1
**UCP-1:**: Uncoupling protein 1
**WAT:**: White adipose tissue
